# Vitamin C supplementation promotes mental vitality in healthy young adults: results from a cross-sectional analysis and a randomized, double-blind, placebo-controlled trial

**DOI:** 10.1007/s00394-021-02656-3

**Published:** 2021-09-02

**Authors:** Minju Sim, Sehwa Hong, Sungwoong Jung, Jin-Soo Kim, Young-Tae Goo, Woo Young Chun, Dong-Mi Shin

**Affiliations:** 1 Department of Food and Nutrition, Seoul National University, 1, Gwanak-ro, Gwanak-gu, Seoul, 08826 Republic of Korea; 2 Seoul W Internal Medicine Clinic, 165, Gwanak-ro, Gwanak-gu, Seoul, 08787 Republic of Korea; 3 Department of Psychology, Chungnam National University, 99, Daehak-ro, Yuseong-gu, Daejeon, 34134 Republic of Korea; 4 Research Institute of Human Ecology, Seoul National University, 1, Gwanak-ro, Gwanak-gu, Seoul, 08826 Republic of Korea; 5 Kwang Dong Pharmaceutical Co., Ltd., 85, Seochojungang-ro, Seocho-gu, Seoul, 06650 Republic of Korea

**Keywords:** Ascorbic acid, Vitamin C supplementation, Mental vitality, Work engagement, Attention, Stroop test

## Abstract

**Purpose:**

We aimed to investigate the link of vitamin C status with vitality and psychological functions in a cross-sectional study, and examine their causal relationship through a randomized controlled trial (RCT).

**Methods:**

We first conducted a population-based cross-sectional investigation of healthy young adults (*n* = 214, 20–39 years), and analyzed the associations of serum vitamin C concentrations with vitality (fatigue and attention) and mood status (stress, depression, and positive and negative affect) using Pearson’s correlation and multiple linear regression analyses. Next, we performed a double-blind RCT in healthy subjects whose serum vitamin C concentrations were inadequate (< 50 μmol/L). Subjects were randomly allocated to receive 500 mg of vitamin C twice a day for 4 weeks (*n* = 24) or a placebo (*n* = 22). We assessed vitality, which included fatigue, attention, work engagement, and self-control resources, and measured mood status, including stress, depression, positive and negative affect, and anxiety. ELISA determined serum brain-derived neurotrophic factor (BDNF), and a Stroop color–word test evaluated attention capacity and processing speed.

**Results:**

In the cross-sectional data, the serum vitamin C concentration was positively associated with the level of attention (*r* = 0.16, *p* = 0.02; standardized *β* = 0.21, *p* = 0.003), while no significant associations with the levels of fatigue and mood variables being found. In the RCT, compared to the placebo, the vitamin C supplementation significantly increased attention (*p* = 0.03) and work absorption (*p* = 0.03) with distinct tendency of improvement on fatigue (*p* = 0.06) and comprehensive work engagement (*p* = 0.07). The vitamin C supplementation did not affect mood and serum concentrations of BDNF. However, in the Stroop color–word test, the subjects supplemented with vitamin C showed better performance than those in the placebo group (*p* = 0.04).

**Conclusion:**

Inadequate vitamin C status is related to a low level of mental vitality. Vitamin C supplementation effectively increased work motivation and attentional focus and contributed to better performance on cognitive tasks requiring sustained attention.

**Trial registration number and date of registration:**

Cross-sectional study: KCT0005074 (cris.nih.go.kr)/1 June, 2020 (retrospectively registered). Intervention study: KCT0004276 (cris.nih.go.kr)/4 September, 2019.

**Supplementary Information:**

The online version contains supplementary material available at 10.1007/s00394-021-02656-3.

## Introduction

Vitamin C (ʟ-ascorbic acid or ascorbate) is an essential nutrient in humans that functions as an indispensable electron donor and a cofactor in various biological reactions such as hydroxylation of collagen, biosynthesis of carnitine, and tyrosine metabolism [1]. Interestingly, vitamin C presents its highest concentrations in the brain [2], and animal model and in vitro studies have reported that vitamin C performs critical roles in brain functions. Vitamin C protects neurons from oxidative stress, induces differentiation and maturation of neurons, and regulates the synthesis or release of neuro-modulating factors including serotonin, catecholamines, and glutamate [3, 4]. Accordingly, vitamin C is inferred to be important for maintaining normal mental health.

Humans rely on dietary supply to obtain vitamin C due to the absence of a gene encoding ʟ-gulonolactone oxidase, which is critical for vitamin C synthesis from glucose [5]. Although vitamin C deficiency can be prevented by consuming one or two servings of citrus fruits or vegetables; reports state that an inadequate vitamin C status is prevalent among young adults even in industrialized countries [6, 7]. The poor vitamin C status in the young can be attributed to external factors such as smoking, excessive drinking, and unhealthy eating habits that fail to provide a fresh and balanced diet rich in vitamin C [8]. Thus, even healthy young individuals can be at risk of vitamin C deficiency, and consequently, poor body functions due to these lifestyle-related factors. However, compared to the elderly, inadequate vitamin C status in the young is liable to remain undiagnosed or be considered as being of little importance.

Vitality is defined as a subjective feeling of energy and aliveness, which highlights the psychological aspects [9]. Feeling vital is suggested as a key component in healthy psychological functioning, the ability of self-regulation, work performance, and goal achievement [10, 11]. Classically, vitality decline is known as the earliest sign of scurvy, a clinical symptom of severe vitamin C deficiency; it manifests in fatigue, decreases in arousal and motivation, and cognitive impairment [12–15]. Considering that professional and social engagement is highest in the young population, it is necessary to investigate whether improvement of vitamin C status helps to promote their vitality and work performance. However, the link of vitamin C status with vitality-related psychological and cognitive functions at a young age is equivocal and their causal relationship has rarely been examined.

Hence, we first investigated the associations between vitamin C status and subjective vitality in a healthy young population. Next, using a randomized, double-blind, placebo-controlled trial, we further explored the effects of vitamin C supplementation on vitality, such as work motivation and self-regulatory resources, and cognitive performance in young adults with inadequate vitamin C status.

## Methods

### Assessment of the associations of serum vitamin C concentration with fatigue, attention, and mood status: a cross-sectional study

#### Participants

The sample size of the cross-sectional study was determined based on the calculation formula [16] with the prevalence of vitamin C deficiency in young adults previously reported [17], a confidence level of 95%, and a margin of error of 5%. Participants were recruited from Seoul National University from June to August 2018. The inclusion criteria were as follows: young adults aged 20–39 with no acute or chronic disease such as hypertension, diabetes, cardiovascular disease, dyslipidemia, kidney disease, cancer, gastrointestinal disorders, and endocrine disorders. A total of 214 men and women volunteered and were included in the cross-sectional study.

#### Study procedure

All participants attended a laboratory measurement between 0800 and 1000 after a 12-h fast, having avoided excessive exercise and alcohol consumption during the last 24 h. During the visit, their height (cm) and weight (kg) were measured using an automatic digital scale (BSM330; InBody Co., Ltd., Seoul, Korea). The precision of height and weight measurement was to the nearest 0.1 cm and 0.1 kg, respectively. Body mass index (BMI) was calculated as weight divided by the square of height (kg/m^2^). Data on smoking status, alcohol consumption, and vitamin C supplement use were collected with a questionnaire. Physical activities were investigated using the International Physical Activity Questionnaire (IPAQ) [18] and were categorized as low (< median) and high (≥ median). Fatigue and attention were measured using two items each from the checklist individual strength, which is a widely used multidimensional instrument [19] (“I feel tired” and “I tire easily” for fatigue assessment, with Cronbach’s *α* = 0.84; “When I am doing something, I can keep my thoughts on it” and “I find it easy to concentrate” for attention assessment, with Cronbach’s *α* = 0.79). Each item was scored with a seven-point Likert scale anchored by “strongly disagree” (1) and “strongly agree” (7), where higher scores reflected a higher level of fatigue and attention. The levels of stress, depression, and positive and negative affect were assessed with stress response inventory (SRI), Beck’s Depression Inventory (BDI), and positive affect and negative affect schedule (PANAS), respectively. The SRI is composed of 22 items concerning psychological, physiological, and behavioral stress responses [20]. The BDI is one of the most widely used instruments for determining the severity of depression; it consists of 21 questions [21]. The PANAS is a reliable and well-validated instrument measuring both positive (10 items) and negative affect (10 items) [22]. Fasting venous blood samples from the antecubital fossa were collected into 4 mL serum separator tubes (BD Biosciences, Franklin Lakes, NJ, USA) in the resting state. We separated the serum samples via centrifugation without delay, and the supernatant of the serum was aliquoted into 1.5 mL ep tubes (Eppendorf, Hamburg, Germany) and immediately stored at − 80 °C. All measurements of vitamin C concentration were performed within 24 h of the blood collection using a high-performance liquid chromatography kit {Chromsystems Instruments & Chemicals GmbH, Gräfelfing, Germany; intra-assay coefficient of variation [CV] < 2.5%, inter-assay CV < 5%} under the manufacturer’s protocol.

This observational study was performed at the Department of Food and Nutrition at Seoul National University between June and August 2018, and was approved by the Institutional Review Board (IRB) of Seoul National University (1806/002-009). The study was retrospectively registered at the Clinical Research Information Service (CRIS) on June 1st, 2020 (KCT0005074). All procedures were performed in accordance with the relevant guidelines and regulations. Written informed consent was provided by all participants prior to their inclusion in the study.

### Assessment of the effects of vitamin C supplementation on vitality, mood status, and cognitive function: an intervention study

#### Participants

Participants were recruited from September to October 2019 from Seoul National University. Men and women who met the following criteria volunteered for participation: (1) 20–39 years of age; (2) no medical history of acute or chronic diseases such as hypertension, diabetes, cardiovascular disease, dyslipidemia, kidney disease, cancer, gastrointestinal disorders, and endocrine disorders; and (3) no use of vitamin C supplements. Next, an assessment of serum vitamin C concentration was carried out to screen volunteers with inadequate vitamin C status, which was defined as a blood concentration of less than 50 μmol/L [23]. Eligible volunteers attended a screening visit between 0800 and 1000. During the screening, all participants completed anthropometric measurements (body height and weight), followed by blood sample collection. Height and weight were measured using the automatic scale (BSM330; InBody Co., Ltd., Seoul, Korea). Fasting venous blood samples from the antecubital fossa were collected into 4 mL serum separator tubes (BD Biosciences, Franklin Lakes, NJ, USA) to determine the fasting vitamin C concentration. All analyses of vitamin C concentration were conducted within 24 h of collection in the same way as in our previous cross-sectional study.

The sample size of the intervention study was calculated as 50 based on the attention score data of our cross-sectional study with *α* = 0.05 and a power of 80%. Finally, a total of 50 individuals (28 men and 22 women) who had shown a vitamin C concentration of less than 50 μmol/L were included in the vitamin C supplementation trial.

This intervention study was conducted in the Department of Food and Nutrition at Seoul National University between September and December 2019, and was approved by the IRB of Seoul National University (1909/002-011) and was registered at the CRIS on September 4th, 2019 (KCT0004276). All steps were performed in accordance with the relevant guidelines and regulations. Written informed consent was obtained from all participants before their inclusion in the study.

#### Study design

This study was a 4-week, randomized, double-blind, parallel, placebo-controlled trial. A total of 50 participants were randomly assigned to either a vitamin C supplementation group (500 mg of vitamin C twice a day for 4 weeks) (*n* = 25) or a placebo supplementation group (*n* = 25), and the random assignment was stratified by sex using a pseudo-random number generator (http://www.randomizer.org). The supplementation was carried out with 2 different drink conditions: (1) 100 mL of the vitamin C drink contained 50 kcal of energy, 11 g of sugar, 1.2 mg of vitamin B_2_, 30 mg of sodium, and 500 mg of vitamin C; and (2) 100 mL of the placebo drink had the identical nutritional content as the vitamin C drink, except for the vitamin C (0 mg). Participants in both the vitamin C and placebo groups were advised to maintain their usual diet including fruits and vegetables and physical activities, and to avoid consuming any other dietary products fortified with vitamin C. To monitor their dietary intake, participants were asked to complete a 2-day dietary record at the baseline and the endpoint, respectively. The dietary intake was analyzed using CAN-Pro 5.0 (Web ver., Korean Nutrition Society, Korea). Data on smoking status and dietary supplement use were collected using a questionnaire, and physical activity was investigated using IPAQ [18].

#### Intervention

We provided participants with the vitamin C drink and the placebo for 4 weeks. Each drink was packaged in a pouch of 100 mL, and participants were instructed to daily consume the two pouches with a 4-h interval to maximize the intestinal absorption of the vitamin C. The colors and flavors of the two types of drink were identical to blind the participants to their allocation. A label was attached to each pouch, but it included only the participant’s code and the date of manufacturing. All experimental staff and investigators involved in the random assignment, measurement of outcomes, and data analysis were also blinded to the allocation until all analyses were complete. The participants were instructed to record their daily consumption of the drinks, and the records were reviewed weekly to check and encourage their compliance with the trial. All of the experimental drinks were produced and supplied by Kwang Dong Pharmaceutical Co., Ltd.

#### Outcome measurements

A total of two visits were carried out during the intervention: the day before the supplementation started (baseline) and the day after the supplementation was completed (week 4). All participants attended laboratory measurements during the morning hours (i.e., between 0800 and 1000) after a 12-h fast, avoiding excessive exercise and alcohol consumption during the previous 24 h. All assessment was individually conducted in a separate and quiet room under the supervision of the investigator. The schematic diagram of the measurement is shown in Supplementary Figure S1.

##### Vitality

Fatigue, attention, work engagement, and self-control resources were assessed as indicators of vitality. Fatigue and attention were measured using the same questionnaire items as in the cross-sectional study. Work engagement and self-control were assessed using the Utrecht Work Engagement Scale [24] and State Ego Depletion Scale [25], respectively. Work engagement is defined as a positive and fulfilling state of mind toward work, and it can also be applied to the academic activity of a student [26]. The scale includes a total of 17 items categorized into 1 of 3 dimensions (i.e., vigor, dedication, and absorption). The ability of self-control is a trait that can be depleted and replenished [27], which is associated with psychological well-being and better achievement at school or workplace [28]. In this study, five items of the State Ego Depletion Scale were used (e.g., “I feel like my willpower is gone”) [25], and reverse-scored to assess self-control resources.

##### Stroop color–word test

The Stroop color–word test is extensively used to measure cognitive functions such as selective attention, cognitive flexibility, and information processing speed [29]. To assess the sustained attention under the cognitive fatigue and mental stress, a mental arithmetic test was conducted just before the Stroop test [30]. The arithmetic expression included two Arabic numerals and one operator, for example, “five figures + five figures”, “five figures – five figures”, “three figures × double figures”, and “four figures ÷ three figures” [31]. A total of 20 questions were presented and a maximum of one minute was given to calculate each question. There was no significant difference between the groups in the number of correct responses for the mental arithmetic test. Subsequently, a computer-assisted Stroop color–word test was performed, based on a previous study [32]. In an incongruent condition, the words were colored in one of four different colors of ink (blue, red, yellow, and green), and each word was not colored in the respective color (e.g., the word BLUE was not colored in blue ink). Participants were asked to match the corresponding color of the word by pressing the colored keys (blue, red, yellow, and green) as quickly and accurately as possible. The incongruent Stroop color–word test consisted of 128 word items in the 4 trials, which included 32 items each. Stroop test performance was assessed by calculating the sum of the reaction times for the correct responses out of the 128 items [33]. All cognitive tests were programmed in the open-source software Psychopy2 (version 1.81, Peirce, 2007), and the correctness and reaction time of correct responses to the Stroop task were recorded by the program. Each participant completed the assessment under the supervision of a well-trained investigator in a separate and quiet room. After providing sufficient guidance on the cognitive assessment, the investigator did not intervene throughout the examination once it began.

##### Biochemical analysis

Fasting venous blood samples from the antecubital fossa were collected into 8 mL BD Vacutainer^®^ SST^™^ II Advance Plus Blood Collection Tubes (BD Biosciences, San Jose, CA, USA) in the resting state. Serum preparation was performed via centrifugation without delay, and the separated serum samples were aliquoted into 1.5-mL ep tubes and immediately stored at − 80 °C. All analyses of vitamin C concentration were conducted within 24 h of collection in the same method as we had used before. We quantified serum brain-derived neurotrophic factor (BDNF), a protein that regulates neuronal development and function in the nervous system [34], using Human/Mouse BDNF DuoSet ELISA (R&D Systems. Inc., Minneapolis, MN, USA) under the manufacturer’s protocol.

##### Mood status

As secondary outcomes, the levels of stress, depression, and positive and negative affect were assessed with SRI, BDI, and PANAS, respectively. In addition, state anxiety, defined as feelings of nervousness and physiological activation of the autonomic nervous system, was measured with State–Trait Anxiety Inventory-X-1, which consists of 21 questions [35].

### Statistical analysis

All statistical analyses were performed using SPSS version 25.0 for Macintosh (SPSS Inc., Chicago, IL, USA). For the cross-sectional analysis, Pearson’s correlation coefficient was calculated to determine the association of serum vitamin C concentration with the scores of fatigue, attention, stress, depression, and positive and negative affect. Multiple linear regression analyses were performed with the use of each questionnaire score which contained vitality and mood status aspects as a dependent variable and sex, age, BMI, smoking status, physical activity level, alcohol consumption frequency, and serum vitamin C concentration as independent variables. We conducted a per-protocol analysis including only participants who had completed the whole treatment protocol and whose compliance were above 80%. Between-group differences in baseline characteristics were analyzed using an unpaired *t* test for continuous variables and a Pearson’s Chi-square test or Fisher’s exact test for categorical variables. Within-group and between-group differences in dietary intake were analyzed using a paired *t* test and an unpaired *t* test, respectively. A repeated-measures ANOVA with Bonferroni correction was used to determine the time-by-group interaction with time (baseline versus endpoint) as the within-groups factor and treatment (vitamin C versus placebo) as the between-groups factor. A two-way ANOVA determined the group-by-sex interaction effects of vitamin C supplementation on vitality and mood status. A paired *t* test was used for comparisons between the baseline and endpoint (week 4). The comparison of Stroop test performance between the groups was conducted using an unpaired *t* test, and the correlation with the endpoint vitamin C status was determined with a Pearson’s correlation analysis. *P* < 0.05 was considered statistically significant.

## Results

### Serum vitamin C concentration was associated with level of attention

A total of 214 participants (84 men and 130 women; 26.2 ± 3.9 year; 22.2 ± 3.3 kg/m^2^) were tested for their serum vitamin C concentrations in a cross-sectional study (Table 1). The concentrations of serum vitamin C were normally distributed from 16.6 to 164.2 μmol/L with a mean value of 56.0 ± 17.6 (Supplementary Figure S2 and Table 1).

**Table 1:** Characteristics of participants included in the cross-sectional study

Characteristics	Total (*n* = 214)	Men (*n* = 84)	Women (*n* = 130)
Age, years	26.2 ± 3.9	26.4 ± 3.8	26.1 ± 3.9
Height, cm	167.0 ± 7.5	174.1 ± 5.3	162.4 ± 4.6
Weight, kg	62.5 ± 13.0	73.9 ± 11.5	55.1 ± 7.5
BMI, kg/m^2^	22.2 ± 3.3	24.3 ± 3.3	20.9 ± 2.4
Current smoker, *n* (%)	14 (6.5)	9 (10.7)	5 (3.8)
Physical activity level			
Low, *n* (%)	106 (49.5)	31 (36.9)	75 (57.7)
High, *n* (%)	108 (50.5)	53 (63.1)	55 (42.3)
Alcohol user, *n* (%)	176 (82.2)	74 (88.1)	102 (78.5)
Vitamin C supplement users, *n* (%)	60 (28.0)	29 (34.5)	39 (30.0)
Serum vitamin C concentration, μmol/L	56.0 ± 17.6	51.4 ± 19.1	58.9 ± 15.9
Vitality			
Fatigue	8.9 ± 2.2	8.7 ± 2.1	9.1 ± 2.2
Attention	7.8 ± 2.0	8.0 ± 2.0	7.7 ± 2.0
Mood			
Stress	40.6 ± 26.7	33.1 ± 23.3	45.4 ± 27.7
Depression	7.6 ± 6.5	5.4 ± 5.0	9.0 ± 6.9
Positive affect	18.8 ± 7.6	20.5 ± 7.5	17.8 ± 7.5
Negative affect	9.7 ± 6.7	8.5 ± 6.2	10.5 ± 6.9

Values are mean ± SD or categorical total

We assessed the subjects’ mood status, and determined the relationships with vitamin C status. Serum vitamin C concentrations did not show significant associations with scores of stress, depression, and positive and negative affect (All *p* > 0.05) (Supplementary Table S1).

As indicators of vitality, the levels of fatigue and attention were assessed. The score of fatigue was not significantly correlated with the serum concentration of vitamin C in either the Pearson’s correlation (*r* = − 0.07, *p* = 0.34) (data not shown) or multiple linear regression analyses (Table 2). On the other hand, Pearson’s correlation analysis revealed that the attention score was positively correlated with the serum concentration of vitamin C (*r* = 0.16, *p* = 0.02) (data not shown). The association between attention scores and serum concentrations of vitamin C was significant when controlling for sex, age, BMI, smoking status, physical activity level, and alcohol consumption frequency with the multiple linear regression model (standardized *β* = 0.21, *p* = 0.003) (Table 2).

**Table 2:** Multiple linear regression analysis for the association between serum vitamin C concentration and subjective vitality in the cross-sectional population (*n* = 214)

Independent variables	Fatigue			Attention		
	*β* (SE)	Standardized *β*	*P*	*β* (SE)	Standardized *β*	*P*
Serum vitamin C concentration (μmol/L)	− 0.01 (0.01)	− 0.10	0.13	0.02 (0.01)	0.21	0.003
Women vs. men	0.36 (0.36)	0.08	0.31	− 0.42 (0.33)	− 0.10	0.21
Age (years)	0.12 (0.04)	0.21	0.003	− 0.08 (0.04)	− 0.15	0.03
BMI (kg/m^2^)	− 0.08 (0.05)	− 0.12	0.14	0.02 (0.05)	0.04	0.64
Smokers vs. non-smokers	1.15 (0.60)	0.13	0.06	0.25 (0.56)	0.03	0.65
High physical activity vs. low^a^	− 0.08 (0.31)	− 0.02	0.81	0.09 (0.29)	0.02	0.74
Alcohol users vs. non-users	0.05 (0.39)	0.01	0.90	0.24 (0.36)	0.05	0.51

*R*^2^ value for fatigue and attention was 0.79 and 0.07, respectively

^a^Physical activities were categorized as low (< median) and high (≥ median)

The observation that vitamin C status was associated with the level of attention prompted us to examine the causal link between vitamin C status and various neuropsychological functions related to vitality in a randomized, double-blinded, placebo-controlled trial.

### Characteristics of the intervention study population

**Fig. 1:**
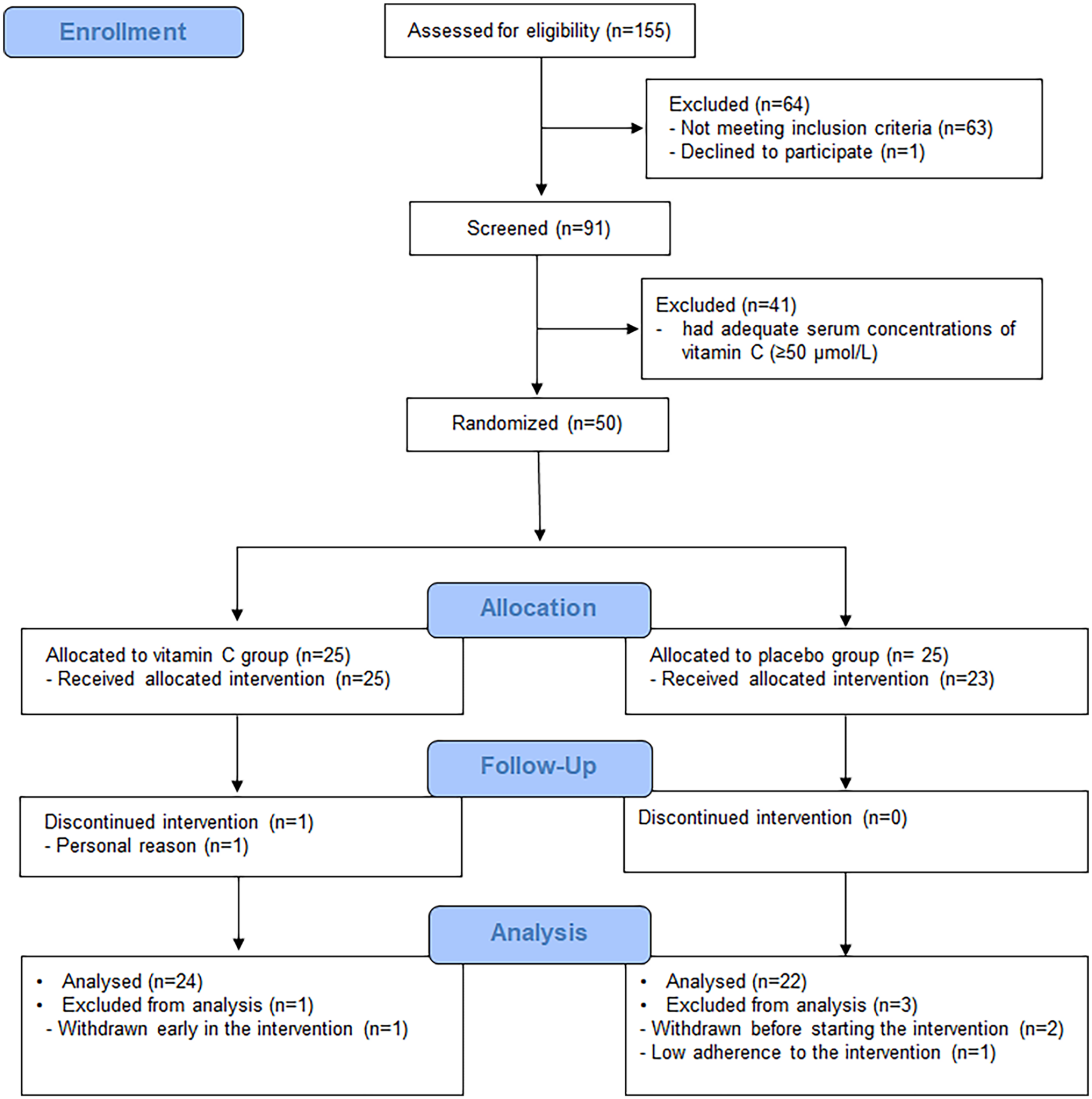
Flowchart of participation throughout the course of the intervention trial

A total of 155 volunteers were assessed for eligibility based on the inclusion criteria (Fig. 1). As a result, 91 subjects were screened to determine their serum vitamin C concentration. Finally, 50 participants with serum vitamin C concentrations lower than 50 μmol/L were included in the intervention study and randomly assigned to either the vitamin C supplementation group (*n* = 25) or the placebo group (*n* = 25) (Fig. 1). A total of four participants were excluded from the final analysis due to the withdrawal of participation or low adherence to treatment (Fig. 1). The baseline characteristics of the participants are summarized in Table 3. There were no significant differences between the vitamin C group and the placebo group in age, sex ratio, BMI, proportions of occupations, current smokers and dietary supplement users, and physical activity (all *p* > 0.05) (Table 3). The vitamin C concentrations at baseline were 42.5 ± 11.7 and 40.2 ± 17.2 μmol/L in the vitamin C and the placebo groups, respectively, with no significant group-difference (*p* = 0.59; unpaired *t* test) (Table 3). At the baseline, the average daily intake of vitamin C did not differ between the groups (*p* = 0.46; unpaired *t* test) (Table 3). The endpoint dietary intake of energy and nutrients including vitamin C was not significantly different compared with those at the baseline in both groups, and there were no significant differences for the change in dietary intake between the vitamin C and the placebo groups (all *p* > 0.05) (Supplementary Table S2).

**Table 3:** Participants’ general characteristics of the intervention study (*n* = 46)

	Vitamin C			Placebo			*P* value
	Total	Men	Women	Total	Men	Women	
No. of subjects	24	14	10	22	12	10	0.79
Age (year)	24.6 ± 3.5	24.9 ± 3.6	24.1 ± 3.5	23.7 ± 1.9	24.0 ± 2.0	23.3 ± 1.8	0.27
BMI (kg/m^2^)	22.6 ± 3.0	24.2 ± 2.6	20.3 ± 1.9	22.3 ± 3.3	23.5 ± 2.0	20.8 ± 4.1	0.74
Occupation							0.89
Undergraduate student (*n*)	17	10	7	17	9	8	
Graduate student (*n*)	3	2	1	3	2	1	
Employee (*n*)	4	2	2	2	1	1	
Current smoker (*n*)	1	1	0	3	2	1	0.60
Dietary supplement user (*n*)	3	3	0	2	2	0	> 0.99
Protein	2	2	0	1	1	0	
Vitamin D	1	1	0	1	1	0	
Physical activity (MET-min/week)	2057 ± 1369	2219 ± 1205	1831 ± 1612	1889 ± 1379	2022 ± 1354	1730 ± 1465	0.68
Serum vitamin C concentration (μmol/L)	42.5 ± 11.7	41.5 ± 12.7	43.9 ± 10.6	40.2 ± 17.2	35.0 ± 10.8	46.4 ± 21.6	0.59
Daily vitamin C intake (mg/day)	41.3 ± 43.8	53.2 ± 14.0	24.7 ± 17.0	52.5 ± 57.8	48.1 ± 43.6	57.8 ± 73.6	0.46

Values are mean ± SD or categorical total

*P* values were obtained by comparing the vitamin C group (*n* = 24) with the placebo group (*n* = 22) using an unpaired *t* test, a Pearson’s Chi-square test, or a Fisher’s exact test

### Vitamin C supplementation successfully increased the serum concentration of vitamin C

**Fig. 2:**
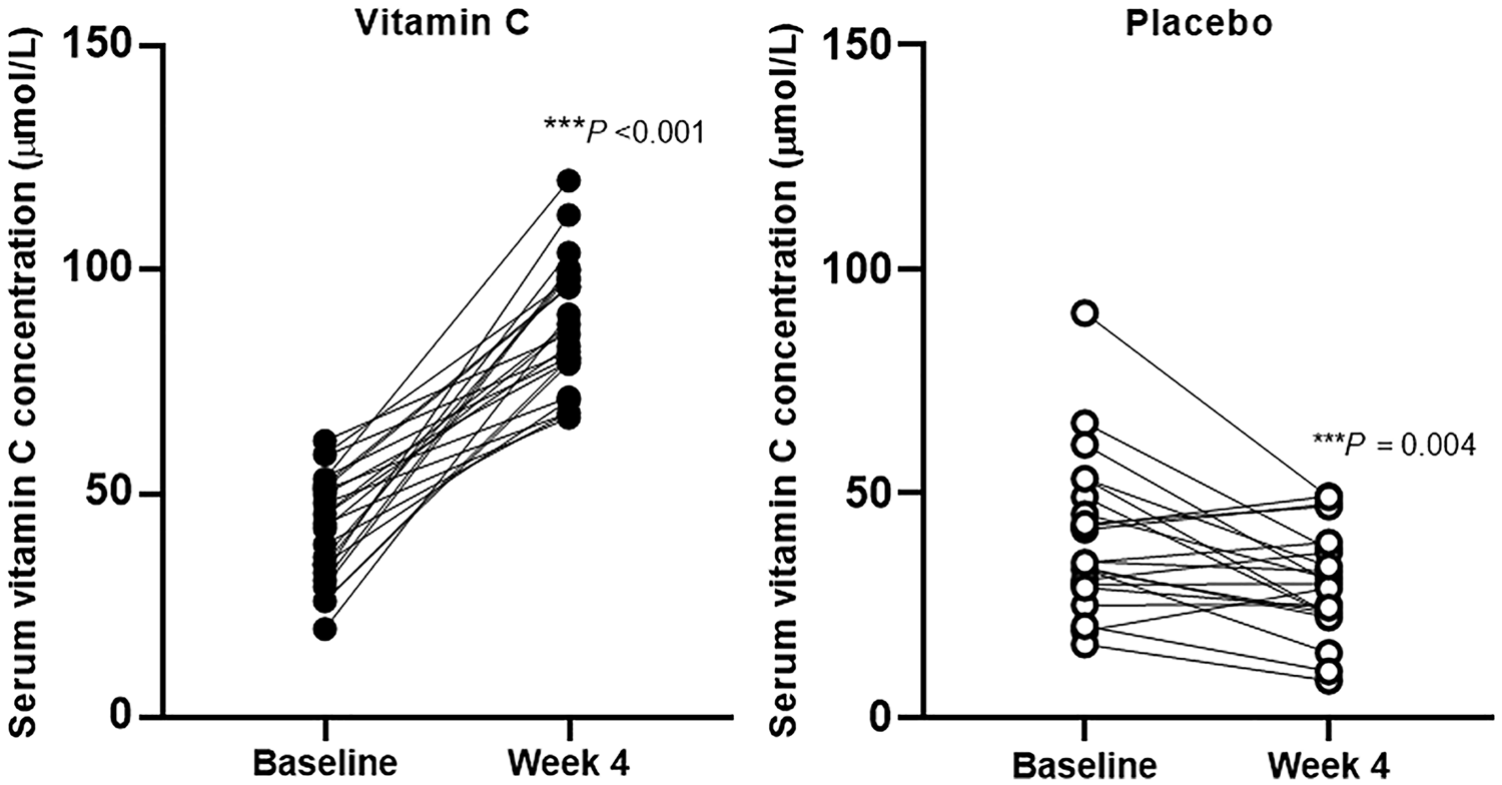
Effect of vitamin C supplementation on serum concentrations of vitamin C. *P* values were obtained using a paired *t* test

After 4 weeks of the intervention, the vitamin C group showed a dramatic increase in serum vitamin C concentration (∆ = 45.17 ± 18.90 μmol/L) (*p* < 0.001; paired *t* test) (Fig. 2). All participants in the vitamin C group had over 50 μmol/L, indicative of optimal status [23]. On the other hand, the placebo group did not show an increase in vitamin C concentration compared to baseline (Fig. 2).

### Vitamin C supplementation increased attention and absorption in work

In the intervention study, we assessed the level of fatigue, attention, work engagement, and self-control resources as indicators of vitality (Table 4). A repeated-measures ANOVA revealed that the vitamin C group showed greater increases in scores of attention and work absorption than the placebo group (both *p* = 0.03) (Table 4). There were distinct trends toward significance for a decrease in fatigue (*p* = 0.06) and an increase in comprehensive work engagement (*p* = 0.07) in the vitamin C group (Table 4). On the other hand, there was no significant treatment effect on self-control resources (*p* = 0.72) (Table 4). No significant effects of vitamin C were seen on stress, depression, positive and negative affect, or state anxiety (all *p* > 0.05) (Supplementary Table S4). There were no group-by-sex interaction effects of vitamin C supplementation on vitality and mood status (all *p* > 0.05) (Supplementary Table S3 and Table S5).

**Table 4:** Effect of vitamin C supplementation on subjective vitality

	Vitamin C (*n* = 24)			Placebo (*n* = 22)			Vitamin C vs. placebo	
	Baseline	Endpoint	Change^a^	Baseline	Endpoint	Change	Difference in change (95% CI)	*P*^b^
Fatigue	9.3 ± 2.4	7.8 ± 2.7	− 1.5 ± 2.5**	8.9 ± 2.1	8.9 ± 1.9	− 0.05 ± 2.6	− 1.5 (− 3.0, 0.1)	0.06
Attention	7.1 ± 1.8	9.0 ± 2.4	1.9 ± 2.7**	7.7 ± 2.1	8.0 ± 2.0	0.3 ± 2.5	1.6 (0.1, 3.2)	0.03
Work engagement	68.7 ± 14.9	74.4 ± 16.1	5.8 ± 10.2*	74.3 ± 13.9	74.7 ± 14.1	0.4 ± 9.7	5.3 (− 0.6, 11.3)	0.07
Vigor	23.2 ± 5.7	25.8 ± 6.0	2.5 ± 4.2**	25.4 ± 5.4	27.0 ± 5.7	1.5 ± 4.0	1.0 (− 1.4, 3.4)	0.41
Dedication	23.0 ± 6.8	24.1 ± 6.6	1.0 ± 4.0	24.5 ± 5.3	23.9 ± 5.4	− 0.6 ± 4.1	1.7 (− 0.7, 4.1)	0.16
Absorption	22.4 ± 4.9	24.6 ± 5.5	2.2 ± 4.0*	24.3 ± 5.1	23.8 ± 5.9	− 0.5 ± 4.1	2.7 (0.3, 5.1)	0.03
Self-control resources	16.5 ± 4.3	18.5 ± 5.3	2.0 ± 4.7^*^	16.1 ± 4.1	17.5 ± 4.4	1.4 ± 4.8	0.5 (− 2.3, 3.3)	0.72

Values are presented as mean ± SD

There were no significant differences between the vitamin C group and the placebo group for all variables measured at the baseline (all *p* > 0.05; unpaired *t* test)

^a^Baseline and endpoint (week 4) measures differed significantly within the vitamin C group (*p < 0.05, **p < 0.01; paired *t* test)

^b^A repeated-measures ANOVA with Bonferroni correction was used to determine the time-by-group interaction with time as the within-subject factor and treatment (vitamin C versus placebo) as the between-subject factor

### Vitamin C supplementation boosted cognitive performance

**Fig. 3:**
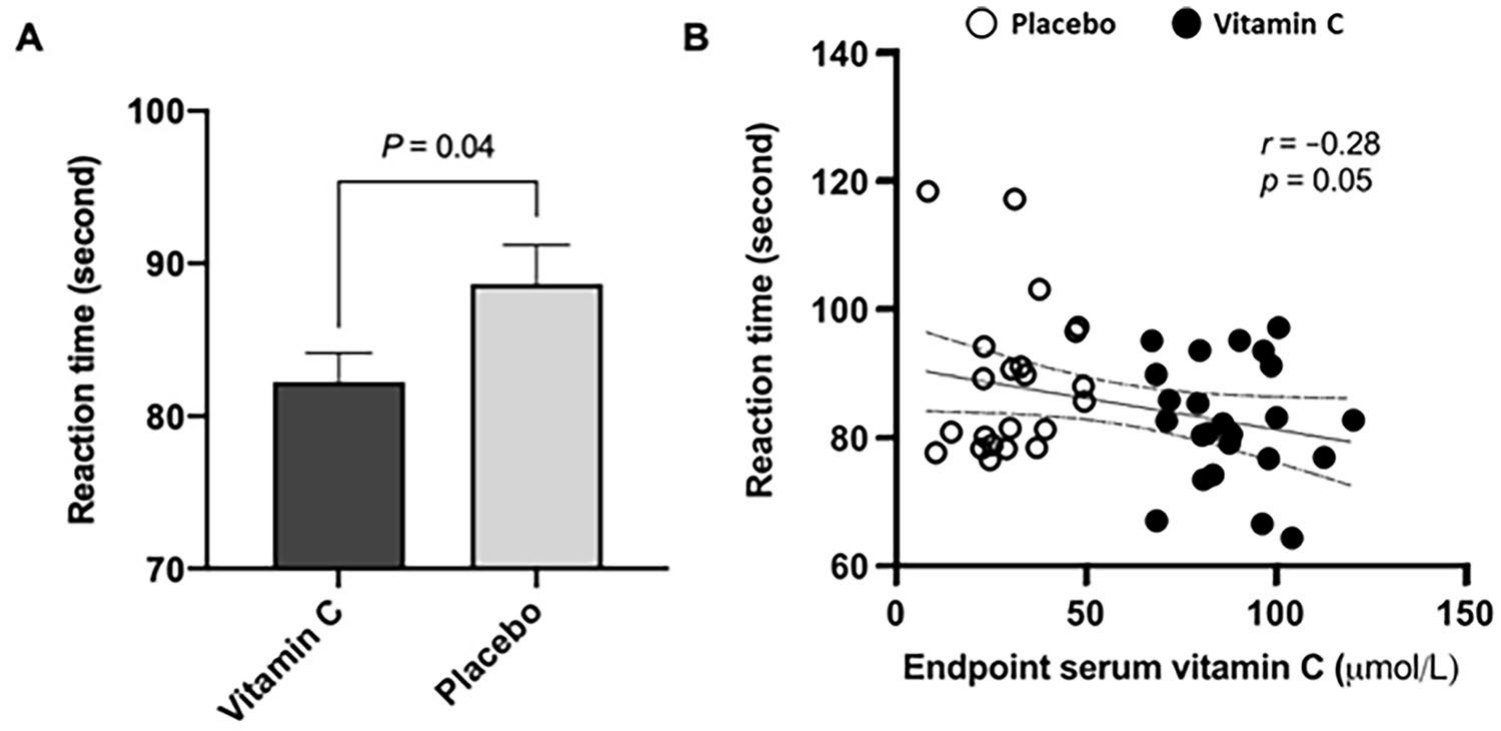
Performance difference in the Stroop color–word test between the vitamin C group (*n* = 24) and the placebo group (*n* = 22). Reaction time was calculated as the sum of the time taken to report the correct answers out of the 128 items. **A** There was a significant difference in the reaction time between the vitamin C and the placebo groups (*p* = 0.04; unpaired *t* test). **B** There was a clear tendency to significance in a correlation between the endpoint serum concentration of vitamin C and the reaction time (*r* = − 0.28, *p* = 0.05; Pearson’s correlation analysis). The correlation plot is shown with a regression line and 95% confidence interval

The Stroop color–word test was performed at week 4 to evaluate sustained attention and processing speed after inducing the mental stress using a mental arithmetic test. While the number of correct responses did not significantly differ between the two groups (*n* = 125.8 ± 2.5 for the vitamin C group; *n* = 126.3 ± 1.0 for the placebo group) (data not shown), in terms of time taken to find the correct answer in the incongruent condition, the vitamin C group showed a significantly shorter reaction time than the placebo group (*p* = 0.04; unpaired *t* test) (Fig. 3A). In addition, there was a clear tendency to significance in a correlation between the endpoint serum concentration of vitamin C and the reaction time (*r* = − 0.28, *p* = 0.05; Pearson’s correlation analysis) (Fig. 3B).

For serum concentrations of BDNF, no significant treatment effect was observed (*p* > 0.05; repeated-measures ANOVA) (Supplementary Figure S3).

## Discussion

To the best of our knowledge, this study is the first to show a relationship between serum vitamin C concentration and neuropsychological performance in healthy adults using both cross-sectional data and a randomized placebo-controlled trial. Population-based investigations have shown serum concentrations of vitamin C to be positively correlated with the level of attention. In a randomized, double-blind, placebo-controlled trial, daily supplementation of 1000 mg vitamin C for 4 weeks stimulated greater absorption in work or study than placebo supplementation in young subjects with below-saturation levels of vitamin C. Moreover, compared to placebo supplementation, vitamin C supplementation significantly increased subjective feeling of concentration and promoted better cognitive performance requiring sustained attention.

Classically, early clinical studies have reported fatigue, mood disturbance, and reduced arousal or motivation in human subjects with severe deficiency or nearly depleted vitamin C status [13, 36, 37]. While the effects of vitamin C status on body function in old age or patients have been intensively studied, there is little information on the relationship between poor vitamin C status and impaired psychological well-being in a healthy young population. We have shown that serum concentrations of vitamin C were positively correlated with the level of attention that indicates mental vitality. Moreover, such a significant association was valid in the multiple linear regression analysis after adjustment for various confounding factors. In particular, vitamin C status showed a stronger effect on attention level than other independent variables such as sex, age, BMI, smoking status, alcohol consumption frequency, and physical activity. Although we did not discover significant associations of vitamin C status with fatigue level and mood status, a few previous studies have reported lower fatigue and better mood in those with higher vitamin C status [38, 39]. Based on our findings, we sought to gain more in-depth insight using an elaborately designed intervention trial. We hypothesized that improved vitamin C status would function as a booster of mental vitality, ultimately promoting cognitive performance.

We supplemented healthy adults’ diets for 4 weeks with either a high dose of vitamin C (1000 mg/day) or a placebo, and evaluated the effects of supplementation on their subjective vitality and cognition. Although the definition of optimal vitamin C status remains to be determined, recent epidemiological studies have acknowledged that 50 μmol/L or higher is adequate to achieve saturation of the body pool [40–42]. In the present study, vitamin C supplementation sharply increased the mean value of serum vitamin C concentration by 106%, and the vitamin C group thoroughly turned into the optimal vitamin C status of 50 μmol/L or higher. To draw reliable findings on the vitamin C supplementation effect, it is necessary to determine the baseline biochemical level of vitamin C and to establish inadequate vitamin C status as an inclusion criterion [40]. It is also important to evaluate whether an improvement in the vitamin C status was achieved after completing the intervention. Our current observation that inadequate vitamin C levels improved optimally after vitamin C supplementation strongly supports a causal relationship between vitamin C status and the promotion of mental functions.

The current study found that vitamin C supplementation induces changes in various psychological measures that have not been reported yet. Most interestingly, the vitamin C group showed a large increase in work absorption, which suggests an increase in motivation for job activities, whereas little change was found in the placebo group. Furthermore, vitamin C supplementation enhanced attentional focus more than in the placebo group, which might have contributed to promoting feelings of being concentrated and engrossed in work [43]. In addition, although we did not find a significant association between fatigue level and vitamin C status in the cross-sectional study, vitamin C supplementation has shown close to significant effect in decreasing fatigue. The fatigue-reducing effect of vitamin C has been reported in previous clinical trials [44–46]. We also examined the effect of vitamin C supplementation on self-regulation resources; there was no treatment effect, although the vitamin C group showed a significant increase compared to the baseline measure. In contrast, in a mouse model genetically modified to be incapable of synthesizing ascorbic acid, vitamin C-deficient mice showed hedonic eating despite the absence of an energy deficit. This observation suggests that vitamin C might act as a physiological substrate for self-regulation [47]. Further studies in human populations are required for further evidence regarding the relationship between vitamin C status and psychological processes such as motivation and self-regulation.

In line with the desirable increase in self- reported vitality, the vitamin C group also exhibited excellent ability on cognitive performance requiring sustained attention: vitamin C group spent much less time finding the correct ink color of the word than the placebo group in the incongruent Stroop color–word test. Moreover, a higher serum concentration of vitamin C was associated with higher performance on the Stroop test in agreement with a previous observational study [48]. This indicates that vitamin C might have contributed to the participants’ staying focused even when significant cognitive load was imposed. BDNF, an essential regulator in the survival of neurons and synaptic plasticity, has been implicated in cognitive functions such as learning and memory in the adults. Evidence from in vitro studies and animal models has linked the contribution of vitamin C to the expression and production of BDNF [49–51]; however, we did not discover the change in BDNF in vitamin C supplementation group. This presents that the vitamin C group’s excellent cognitive performance might have been attributed to an increase in mental vitality rather than improvement of other cognitive domains.

A possible explanation for the increase in mental vitality in the present study is that vitamin C has modulating effects on neurotransmitters and hormones in the brain. In particular, vitamin C interacts with the dopaminergic system: vitamin C mediates the catalyzing of dopamine-β-hydroxylase [52–55], and dopamine signaling regulates vitamin C release and bioavailability in neurons [56]. Dopaminergic signaling is deeply concerned with both emotional arousal (e.g., motivation, goal seeking and self-control) and successful executive control (e.g., attention and cognitive flexibility) [57–60], which were distinctly observed in the current vitamin C supplementation group. Previous animal studies have reported vitamin C deficiency as contributing to abnormalities in the dopaminergic system, such as decreases in dopamine metabolites and locomotor activities and alteration of social behavior [47, 61]. However, to the best of our knowledge, there have been few reports on the efficacy of vitamin C supplementation on the neurotransmitter in humans. Therefore, there is a need to investigate this in-depth in future studies.

There is a growing body of literature that associates vitamin C status with psychological mood. However, we failed to show cross-sectional associations or a supplementation effect of vitamin C on mood variables, including stress, depression, positive and negative affect, and anxiety. In contrast, supplementation with 3000 mg/day of vitamin C lowered subjective stress against acute psychological stressors [62], and a relief effect on anxious moods was observed after vitamin C administration in healthy populations [63, 64].

The limitations of the current study include a relatively short-term intervention period, which prevented us from discerning the treatment effects on several mood variables. Therefore, additional vitamin C supplementation trials with longer durations may help to determine other potential outcomes regarding psychological functions. Furthermore, it is necessary to follow-up the changes in neurotransmitters, neurohormones, and neurotrophins in further studies to understand the underlying mechanisms by which vitamin C affects brain functions.

In conclusion, this study is the first, to our knowledge, to show the link between vitamin C status with mental functions in healthy young adults using both population-based observational studies and randomized clinical trials. The cross-sectional study suggests inadequate vitamin C status is related to a low level of mental vitality. In the randomized clinical trial, vitamin C supplementation at 1000 mg/day for 4 weeks effectively increases serum vitamin C concentrations in subjects with suboptimal vitamin C status. The supplementation promotes their mental vitality, especially work motivation and attentional focus, contributing to better performance on cognitive tasks that require sustained attention.

## Supplementary Information

Below is the link to the electronic supplementary material.

Supplementary file1 (DOCX 151 KB)

Supplementary file2 (XLSX 54 KB)

## Data Availability

The datasets generated during the current study are available from an electronic supplementary material.

## Abbreviations

SRI: Stress response inventory
BDI: Beck’s Depression Inventory
PANAS: Positive affect and negative affect schedule
BDNF: Brain-derived neurotrophic factor
