# Supplementary material for: Vitamin C supplementation promotes mental vitality in healthy young adults: results from a cross-sectional analysis and a randomized, double-blind, placebo-controlled trial
Source: Eur J Nutr. 2021 Sep 2;61(1):447–59. doi: 10.1007/s00394-021-02656-3 (PMC8783887; doi:10.1007/s00394-021-02656-3)
Supplement: Supplementary file 1 — Supplementary file1 (DOCX 151 KB) [file 394_2021_2656_MOESM1_ESM.docx]

**Table S1**

Pearson’s correlation and multiple linear regression analyses for the association between serum vitamin C concentration and mood status in the cross-sectional population (*n* = 214)

|  | Pearson’s correlation | |  | Multiple linear regression | | | | |
| --- | --- | --- | --- | --- | --- | --- | --- | --- |
|  | *r* | *P* |  | Unstandardized *β* | Standard error | Standardized *β* | *P* | *R^2^* |
| Stress | –0.06 | 0.35 |  | –0.19 | 0.10 | –0.12 | 0.07 | 0.13 |
| Depression | –0.03 | 0.62 |  | –0.04 | 0.02 | –0.11 | 0.09 | 0.18 |
| Positive affect | 0.08 | 0.23 |  | 0.05 | 0.03 | 0.11 | 0.09 | 0.12 |
| Negative affect | –0.05 | 0.49 |  | –0.03 | 0.03 | –0.09 | 0.19 | 0.11 |

Independent variables of multiple linear regression included serum vitamin C concentration (μmol/L), sex (men=0, women=1), age (y), BMI (kg/m^2^), physical activity level (low=0, high=1), current smoking (no=0, yes=1), and alcohol use (no=0, yes=1).

**Table S2**

Average daily intake of energy and nutrients in the intervention study population (*n* = 46)

|  | Vitamin C (*n* = 24) | | |  | Placebo (*n* = 22) | | |  | *P*^a^ |
| --- | --- | --- | --- | --- | --- | --- | --- | --- | --- |
|  | Baseline | Endpoint | Change |  | Baseline | Endpoint | Change |  |  |
| Energy (kcal/d) | 1725.3 ± 444.3 | 1802.1 ± 503.1 | 76.8 ± 611.0 |  | 1763.4 ± 505.0 | 1784.3 ± 351.2 | 20.9 ± 506.3 |  | 0.73 |
| Carbohydrate (g/d) | 221.0 ± 61.3 | 221.9 ± 61.6 | 0.9 ± 98.5 |  | 227.8 ± 72.6 | 222.1 ± 53.5 | −5.7 ± 78.6 |  | 0.80 |
| Protein (g/d) | 66.9 ± 17.8 | 73.2 ± 27.3 | 6.3 ± 31.5 |  | 68.8 ± 23.9 | 72.0 ± 22.3 | 3.2 ± 27.0 |  | 0.72 |
| Fat (g/d) | 60.3 ± 25.5 | 62.6 ± 28.7 | 2.3 ± 33.1 |  | 57.6 ± 21.0 | 60.4 ± 21.2 | 2.8 ± 27.8 |  | 0.95 |
| Cholesterol (mg/d) | 303.6 ± 147.1 | 362.4 ± 168.3 | 58.9 ± 247.6 |  | 347.9 ± 165.9 | 343.4 ± 186.9 | −4.6 ± 226.5 |  | 0.37 |
| Fiber (g/d) | 15.5 ± 5.8 | 15.9 ± 7.1 | 0.5 ± 8.9 |  | 17.2 ± 7.5 | 14.4 ± 5.4 | −2.8 ± 9.2 |  | 0.22 |
| Vitamin C (mg/d) | 41.3 ± 43.8 | 34.1 ± 26.8 | −7.2 ± 56.8 |  | 52.5 ± 57.8 | 39.0 ± 29.6 | −13.5 ± 66.2 |  | 0.73 |

Values are presented as mean ± SD. Baseline and endpoint (week four) measures did not differ significantly within the group (all *p* > 0.05; paired *t* test).

^a^*P* values were obtained comparing the intake change between the groups (all *p* > 0.05; unpaired t test).

**Table S3**

Effect of vitamin C supplementation on subjective vitality by sex

|  | Vitamin C (*n* = 24) | | | | |  | Placebo (*n* = 22) | | | | |  |  | |
| --- | --- | --- | --- | --- | --- | --- | --- | --- | --- | --- | --- | --- | --- | --- |
|  | Men (*n* = 14) | |  | Women (*n* = 10 ) | |  | Men (*n* = 12) | |  | Women (*n* = 10) | |  | Group × Sex | |
|  | Baseline | Endpoint |  | Baseline | Endpoint |  | Baseline | Endpoint |  | Baseline | Endpoint |  | *F* | *P* |
| Fatigue | 9.0 ± 2.5 | 7.1 ± 2.9 |  | 9.6 ± 2.3 | 8.7 ± 2.2 |  | 9.1 ± 1.9 | 9.0 ± 1.9 |  | 8.7 ± 2.4 | 8.7 ± 1.9 |  | 1.46 | 0.23 |
| Attention | 7.0 ± 2.1 | 9.1 ± 2.6 |  | 7.2 ± 1.4 | 8.8 ± 2.3 |  | 7.7 ± 2.5 | 8.3 ± 2.2 |  | 7.8 ± 1.6 | 7.7 ± 1.9 |  | 0.02 | 0.89 |
| Work engagement | 71.9 ± 15.1 | 79.6 ± 16.1 |  | 64.1 ± 13.9 | 67.2 ± 13.8 |  | 77.3 ± 15.4 | 78.6 ± 17.9 |  | 70.7 ± 11.5 | 70.0 ± 5.5 |  | 0.24 | 0.63 |
| Vigor | 23.8 ± 6.6 | 27.4 ± 6.5 |  | 22.4 ± 4.4 | 23.4 ± 4.5 |  | 26.6 ± 6.0 | 27.9 ± 6.9 |  | 24.0 ± 4.4 | 25.8 ± 4.0 |  | 1.48 | 0.23 |
| Dedication | 25.1 ± 6.5 | 26.0 ± 6.3 |  | 20.2 ± 6.3 | 21.4 ± 6.3 |  | 26.0 ± 5.4 | 25.8 ± 6.1 |  | 22.8 ± 4.9 | 21.6 ± 3.4 |  | 0.14 | 0.71 |
| Absorption | 23.1 ± 4.4 | 26.1 ± 5.3 |  | 21.5 ± 5.6 | 22.4 ± 5.2 |  | 24.7 ± 5.7 | 24.8 ± 7.2 |  | 23.9 ± 4.6 | 22.6 ± 3.9 |  | 0.14 | 0.71 |
| Self-control | 17.2 ± 4.7 | 19.3 ± 4.6 |  | 15.5 ± 3.6 | 17.3 ± 6.2 |  | 16.8 ± 4.1 | 17.4 ± 4.9 |  | 15.2 ± 4.0 | 17.7 ± 4.1 |  | 0.70 | 0.41 |

Values are presented as mean ± SD.

The endpoint (week four) measures were compared by a two-way ANOVA with baseline measures as a covariate to determine the group-by-sex interaction effect.

**Table S4**

Effect of vitamin C supplementation on mood status

|  | Vitamin C (*n* = 24) | | |  | Placebo (*n* = 22) | | |  | Vitamin C *vs.* Placebo | |
| --- | --- | --- | --- | --- | --- | --- | --- | --- | --- | --- |
|  | Baseline | Endpoint | Change |  | Baseline | Endpoint | Change |  | Difference in change (95% CI) | *P*^a^ |
| Stress | 18.1 ± 11.3 | 13.4 ± 12.2 | –4.7 ± 10.4*^*^* |  | 18.8 ± 11.5 | 15.3 ± 9.4 | –3.5 ± 10.5 |  | –1.1 (–7.3, 5.1) | 0.71 |
| Depression | 8.1 ± 6.2 | 5.0 ± 5.1 | –3.1 ± 4.2*^**^* |  | 6.9 ± 3.9 | 3.5 ± 3.4 | –3.3 ± 5.2*^**^* |  | 0.2 (–2.6, 3.0) | 0.86 |
| Positive affect | 18.4 ± 7.5 | 20.7 ± 9.3 | 2.3 ± 6.2 |  | 18.2 ± 8.3 | 18.7 ± 9.2 | 0.5 ± 6.6 |  | 1.7 (–2.0, 5.5) | 0.35 |
| Negative affect | 10.2 ± 8.5 | 10.3 ± 8.2 | 0.0 ± 5.9 |  | 10.0 ± 7.5 | 8.8 ± 7.4 | –1.2 ± 4.8 |  | 1.3 (–1.9, 4.5) | 0.43 |
| State anxiety | 43.3 ± 10.6 | 38.1 ± 10.8 | –5.2 ± 14.3 |  | 38.9 ± 10.5 | 36.2 ± 8.4 | –2.6 ± 10.4 |  | –2.6 (–10.1, 4.9) | 0.49 |

Values are presented as mean ± SD.

There were no significant differences between the vitamin C group and the placebo group for all variables measured at the baseline (All *p* > 0.05; unpaired *t* test).

Baseline and endpoint (week four) measures differed significantly within the group (^*^*p* < 0.05, ^**^*p* < 0.01; paired *t* test).

^a^A repeated-measures ANOVA with Bonferroni correction was used to determine the time-by-group interaction with time as the within-subject factor and treatment (vitamin C versus placebo) as the between-subject factor.

**Table S5**

Effect of vitamin C supplementation on mood status by sex

|  | Vitamin C (*n* = 24) | | | | |  | Placebo (*n* = 22) | | | | |  |  | |
| --- | --- | --- | --- | --- | --- | --- | --- | --- | --- | --- | --- | --- | --- | --- |
|  | Men (*n* = 14) | |  | Women (*n* = 10 ) | |  | Men (*n* = 12) | |  | Women (*n* = 10) | |  | Group × Sex | |
|  | Baseline | Endpoint |  | Baseline | Endpoint |  | Baseline | Endpoint |  | Baseline | Endpoint |  | *F* | *P* |
| Stress | 17.3 ± 13.2 | 11.9 ± 10.9 |  | 19.2 ± 8.5 | 15.6 ± 14.1 |  | 17.8 ± 10.6 | 12.8 ± 8.0 |  | 20.0 ± 13.0 | 18.2 ± 10.4 |  | 0.07 | 0.79 |
| Depression | 8.5 ± 7.5 | 5.5 ± 6.2 |  | 7.6 ± 4.0 | 4.4 ± 3.1 |  | 6.1 ± 4.4 | 2.3 ± 1.8 |  | 7.8 ± 3.2 | 5.0 ± 4.3 |  | 1.38 | 0.26 |
| Positive affect | 19.3 ± 7.4 | 23.1 ± 9.5 |  | 17.1 ± 7.9 | 17.3 ± 8.2 |  | 18.2 ± 9.3 | 18.1 ± 10.9 |  | 18.1 ± 7.3 | 19.5 ± 7.2 |  | 2.12 | 0.15 |
| Negative affect | 10.1 ± 9.0 | 14.0 ± 10.3 |  | 10.3 ± 8.2 | 12.4 ± 9.4 |  | 9.9 ± 7.1 | 7.8 ± 6.4 |  | 10.1 ± 8.2 | 10.0 ± 8.6 |  | 0.24 | 0.62 |
| State anxiety | 40.4 ± 11.5 | 39.0 ± 12.5 |  | 47.4 ± 8.1 | 36.8 ± 8.3 |  | 37.1 ± 10.2 | 35.9 ± 8.2 |  | 41.0 ± 10.9 | 36.6 ± 9.1 |  | 0.40 | 0.53 |

All values are presented as mean ± SD.

The endpoint (week four) measures were compared by a two-way ANOVA with baseline measures as a covariate to determine the group-by-sex interaction effect.


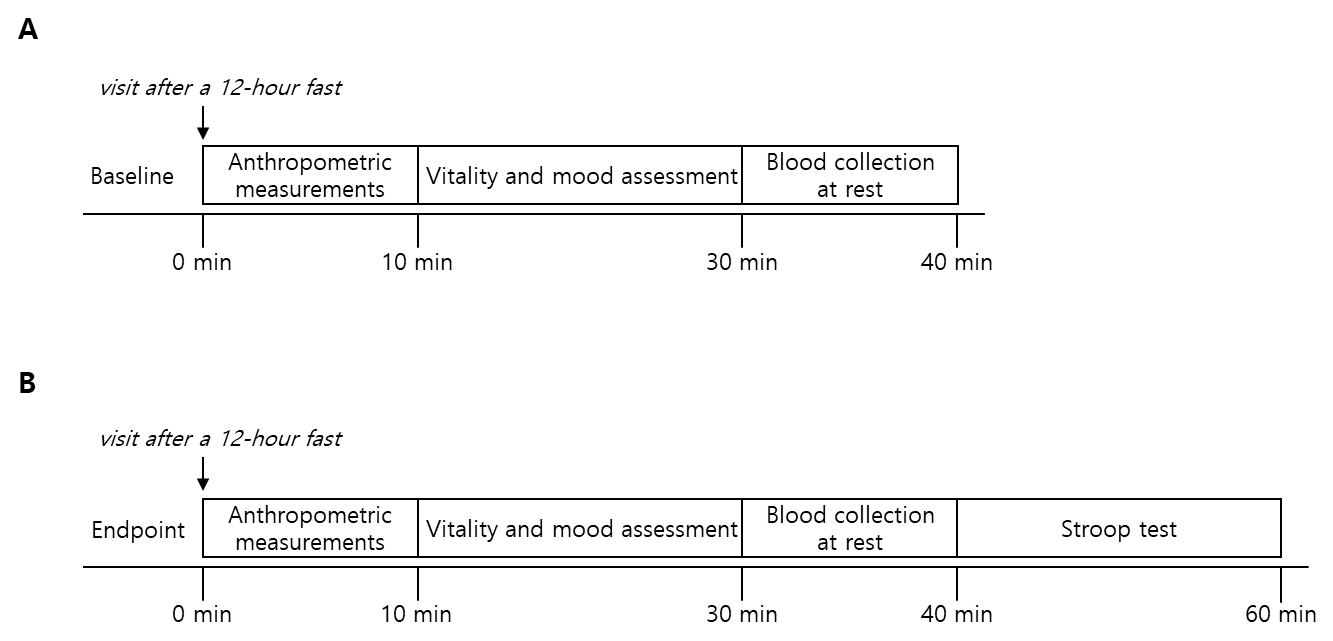


**Figure S1**

Schematic representation of laboratory measurements at the baseline (A) and endpoint (week four) (B). Anthropometric measurements included body height and weight. Vitality and mood assessment consisted of measurements of fatigue, attention, work engagement, self-control, stress, depression, positive and negative affect, and anxiety.

**Figure S2**

Vitamin C distribution in the cross-sectional study population (*n* =214). Normality was tested using Kolmogorov-Smirnov test (*p* = 0.08).

**Figure S3**

Effect of vitamin C supplementation on serum concentrations of brain-derived neurotrophic factor (BDNF). There was no significant difference between the vitamin C group and the placebo group (*p* > 0.05; repeated-measures ANOVA).
